# The Need for Ocular Protection for Health Care Workers During SARS-CoV-2 Outbreak and a Hypothesis for a Potential Personal Protective Equipment

**DOI:** 10.3389/fpubh.2020.599757

**Published:** 2020-11-12

**Authors:** Lixiang Wang, Yingping Deng

**Affiliations:** Department of Ophthalmology, West China Hospital, Sichuan University, Chengdu, China

**Keywords:** SARS-CoV-2, ocular protection, sustained releasing contact lens, personal protective equipment, griffithsin

## Abstract

SARS-CoV-2 is a coronavirus with high infectivity and has caused dramatic pressure on health systems all over the world. Appropriate personal protection for medical staffs is critical. For ocular protection, there is ongoing hot debate and concern for potential ocular transmission of SARS-CoV-2. Ocular manifestations and positive detection of viral RNA in ocular samples were only reported in very small number of patients infected with SARS-CoV-2. However, health care workers need to face patients more closely and have higher risk of aerosol contamination. Thus, appropriate ocular protection for medical workers is still recommended by organizations such as WHO and American Academy of Ophthalmology. Although eye goggles provide excellent protection and are mandatory for medical practitioners with high risk of exposure, they are not ideal for common clinical practice, because they can disturb vision due to extensive formation of water droplets and frequently cause moderate to severe discomfort after longtime wearing, which have been reported to interfere with working status. For the majority of medical workers who don't deal with high risk patients, they are not advised to wear goggles in daily practice. However, they also face the risk of infection due to the presence of asymptomatic carriers. Especially in situations with high risk of ocular exposure, such as close physical examination, eye surgery, dental clinics and surgery, ocular protection may be needed. Griffithsin has been shown to directly bind to spike proteins and has anti-viral activity against a broad spectrum of viruses, including coronavirus. Griffithsin is found to inhibit the entry of SARS-CoV at relatively low concentration and is stable and non-toxic. SARS-CoV-2 and SARS-CoV share the same entry receptors and their spike proteins are similar in conformation. We hypothesize that contact lenses containing nanoparticles loaded with griffithsin may provide sufficient ocular protection for medical staffs without high risk of exposure during the outbreak period of SARS-CoV-2. If proven effective, griffithsin-loaded contact lens can be considered as a supplementary ocular protective equipment for medical workers who can tolerate well. The daily disposable contact lens should be applied as needed and refrain from extended wearing in order to reduce potential side effects.

## Introduction

### The Risk of Ocular Transmission of SARS-CoV-2 in Health Care Workers

The novel coronavirus “SARS-CoV-2” is now causing global pandemic and has claimed more than 800,000 lives until July, 2020. Although SARS-CoV-2 is principally a respiratory virus, there is concern that the ocular surface may serve as potential route of SARS-CoV-2 transmission.

The entry of SARS-CoV-2 into host cells relies on protein-protein interaction of its spike protein (S protein) with host surface receptors (ACE2 or CD147) (1, 2). The critical motif for receptor recognition and binding is found in receptor binding domain (RBD) of S protein. After binding, proteolytic cleavage by membrane protease TMPRSS2 is needed to allow for fusion of virus and cell membrane and subsequent entry, a process called “protein priming”(3, 4). Therefore, ACE2/CD147 and TMPRSS2 are all essential for virus entry and transmission. Previous studies have illuminated that ACE2, TMPRSS2, and CD147 were all expressed on ocular surface, including cornea and conjunctiva (5–7). Thus, theoretically the eye can serve as the entry route for SARS-CoV-2, as this area is likely to be contaminated by aerosol, droplets or direct touching (8). In addition, the ocular surface is anatomically connected with the respiratory tract *via* the nasolacrimal duct. The nasolacrimal duct drains tear in the conjunctival sac continuously into the inferior nasal meatus and is thought to play important roles in the spreading of ocular virus into the respiratory system. Thus, the eye theoretically possesses dual routes for virus spread: lacrimal drainage-based spread and direct infection *via* ocular cell receptors (9). Although currently no evidence of intraocular infection of SARS-CoV-2 is available, some common beta coronaviruses can penetrate inside and lead to retinitis and uveitis (10). Besides, the special region of limbus also provide potential routes for the spread of virus *via* blood circulation or trigeminal nerve branches (11). In one animal study of SARS-CoV-2 on rhesus monkeys, virus inoculated on conjunctival surfaces caused characteristic interstitial pneumonia and was detected in a variety of organs by autopsy (12). Thus, these evidence indicates ocular surface has the structural and physiological foundation for SARS-CoV-2 infection.

However, according to clinical data and RT-PCR tests of ocular samples, ocular involvement and positive isolation of viral RNA were only reported in a very small number of patients infected with SARS-CoV-2 (Tables 1, 2). The main ocular manifestations were symptoms related to conjunctivitis. The reported rates of patients with ocular symptoms were 1.5%-31.6% in different studies (13–18). Particularly to be noted were some medical staffs who were not protected with goggles when they were exposed to SARS-CoV-2 and became infected (13, 17). Zhang et al. reported an emergency nurse infected with SARS-CoV-2 and tested positive in conjunctival swabs. The patient was protected with N95 mask during the whole practice time but found her goggle dislocated. Conjunctivitis was the initial symptom, and the conjunctival sample was tested positive on the second day and turned negative at day 9 (17). Xia et al. also reported a patient presented with conjunctivitis and watery secretions as initial symptoms and virus was detected at the early phase of infection (16). A large cross sectional study of 535 patients showed that ocular symptoms were present in 5.05% of patients, and the average duration of conjunctivitis was 5.9 days (14). According to a recent systematic review which included 11 studies on the topic of ocular involvement in SARS-CoV-2, 3 patients with conjunctivitis had positive PCR test, 8 patients had positive tear-PCR in the absence of conjunctivitis, and 14 patients with conjunctivitis but were tested negative by RT-PCR (21). These clinical results indicated that for the generally population, the link between ocular involvement and SARS-CoV-2 infection is still controversial. At least the ocular surface is not a major route for SARS-CoV-2 transmission.

**Table 1:** Clinical reports of ocular involvement of SARS-CoV-2.

**References**	**Total number of patients**	**Number of patients with ocular symptoms**	**Ocular symptoms as the initial presentation**	**Types of ocular manifestations**
Zhou et al. (13)	67	1	1	Conjunctival injection, watery secretion
Chen et al. (14)	535	27	4	Conjunctival injection
Wu et al. (15)	38	12	1	Conjunctival injection, chemosis, epiphora, increased secretion
Xia et al. (16)	30	1	1	Conjunctival injection
Zhang et al. (17)	72	2	1	Conjunctival injection, epiphora
Xu et al. (18)	30	1	1	Eye itching

**Table 2:** Summary of reviewed articles for the detection of SARS-CoV-2 in tears or conjunctival secretions.

**References**	**Participants**	**Demographic characteristics**	**Positive Detection rate**	**Ocular manifestations**	**Detection phase**	**Duration of positive detection**
Fang et al. (19)	32	F:M=1:1Mean age=41 (34–54)	15.6%	None	During admission time	N.A.
Zhou et al. (13)	67	F:M=1.68 Mean age=36 (22–78)	1.5% positive 3.0% suspected	1.5%	During admission time	N.A.
Zhang et al. (17)	72	F:M=1.00 Mean age=59	1.4%	2.8%	Before and during admission time	Conjunctival swab turned positive 1 day after conjunctivitis, and became negative at day 9
Xu et al. (18)	30	F:M=1.14 Mean age=48	0%	3.3%	During admission time	N.A.
Deng et al. (20)	114	F:M=0.84 Mean age=61 (10–88)	0%	0%	During admission time	N.A.
Xia et al. (16)	30	F:M=0.43 Mean age=55 (13–83)	3.3%	3.3%	During admission time	Detected at day 2 and day 4 after the onset of symptoms

It has been well-recognized that the ocular surface possesses a variety of mechanisms to protect from viral infection, which may explain the low rate of ocular involvement and RNA detection. Many mechanical activities, like tearing, blinking and barrier function of eyelid and lashes may all prevent landing of virus-containing droplets on ocular surface (22). In one experiment on model man, particles of 0.6–5.0 μm were emitted from a jet set 20 cm from the nose. The amount of particles landing on ocular surface was only 1/8 to 1/4 of those on lips, which indicated ocular surface is an uncommon landing area for droplets (8). In addition, the ocular surface possesses multiple innate and acquired immune compounds and actions to defend against viral infection, including lactoferrin, β-lysin, secretory IgA, complement, interferons, *etc*.(23).

Although ocular involvement is infrequent in patients infected with SARS-CoV-2, there is evidence for higher risk of ocular transmission for first-line medical workers and the need for ocular protection during high risk procedures. Many procedures such as tracheal intubation, dental surgery and electrocautery generate high concentration of aerosols which may contain the virus and increase the possibility of ocular landing and transmission (24). For ophthalmological surgeons at high risk of ocular transmission, lack of appropriate personal protection results in reduced amount of surgical interventions and potential delay of necessary operations during SARS-CoV-2 outbreak (25). In one previously published study during the outbreak of SARS-CoV, nurses caring for intubated patients who didn't use eye protection had 8 times higher infection rate than those wearing goggles (8 vs. 1%) (26). Thus, we think although ocular involvement is not common in patients infected with SARS-CoV-2, but still can serve as potential transmission route especially for medical workers. The American Academy of Ophthalmology has recognized the risk of ocular transmission in the beginning and called for appropriate eye protection for ophthalmology workers (27).

### Griffithsin Can Block the Entry of Coronavirus and Other Enveloped Viruses

Griffithsin is a small lectin consisting of 121 amino acids and is derived from *Griffithsia* spp. (28). Grifithsin has been found to be able to block the entry of a variety of enveloped viruses, including HIV, MERS-CoV, SARS-CoV and HCV and efficiently inhibit viral entry, because it has high affinity to bind to multiple sites of glycoproteins on the virus envelope (29–32). In the previous efficacy studies, griffithsin has been tested either as prophylactic agents or therapeutic drugs against viral infection and showed high potency (33). In an *in vitro* study, griffithsin was found to prevent cell fusion and cell-to-cell transmission of HIV at a concentration of <1 nM by binding to its envelop protein gp120 (34). In mice models, intra-vaginal application of gel containing 0.1% griffithsin prevented spread of HSV-2 and significantly reduced disease scores (35). Griffithsin is found to specifically bind to monosaccharides (mannose, glucose, and N-acetylglucosamine) and oligosaccharide moieties of glycoproteins of virus, thus can theoretically work on any virus whose surface proteins are glycosylated, such as S protein of coronavirus (32). In addition, one molecule of griffithsin possesses three identical carbohydrate-binding domains (36). On crystal structures, the three binding sites are located in an equilateral triangle, and each possesses an aspartic acid residue which makes extensive contact with saccharides (36). Thus griffithsin is multivalent and can work at low concentration, and the estimated EC_50_ value to block the activity of SARS-CoV is 0.28–0.96 μM (36). On mice inoculated with lethal doses of SARS-CoV, concomitant administration of 5 mg/kg intranasal griffithsin improved survival rate to 100% and dramatically reduced lung injury (32). Based on the the action of griffithsin and previous studies, we can infer that this small peptide can also block the entry of SARS-CoV-2, because the S protein of SARS-CoV-2 and SARS-CoV are similar in conformation and both glycosylated with high-mannose glycan (37–39). Moreover, griffithsin is very stable and resistant to the degradation of protease and detergent (40). *In vitro* and *in vivo* toxicology studies demonstrate that griffithsin has no cytotoxicity (41). In summary, griffithsin is a safe anti-viral agent and has been shown to block the entry of a wide variety of coronavirus. It is reasonable to hypothesize that griffithsin is a good candidate for SARS-CoV-2 prevention, which has been suggested by several researchers (42, 43).

### Sustained-Releasing Therapeutic Contact Lenses

As the ocular surface is continuously exposed to the environment, a prolonged eye protection is needed. Traditional eye drops may not provide sufficient protection due to blinking and drainage by nasolacrimal duct. It is estimated that drugs administrated via eye drops only reside in tears for 1–3 min and have very low bioavailability (44). Thus, sustained-releasing therapeutic contact lenses containing griffithsin may be the optimal option for the protection of ocular surfaces against SARS-CoV-2. As griffithsin is a small protein, it can be entrapped in nanoparticles which can enable sustained delivery. The technique was first describe by Gulsen et al. who dispersed drug-laden nanoparticles in hydroxyethyl methylacrylate (HEMA) monomers before polymerization to make therapeutic contact lenses (45). The contact lenses containing drug-laden nanoparticles are able to release drugs for an extended period of time, and show reasonably good tolerability, transparency and permeability (46).

## The Hypothesis

The ocular surface is a possible transmission route of SARS-CoV-2, especially for medical staffs who work in close contact with infected patients. Theoretically, griffithsin can bind to S protein on virus envelop and inhibit the entry of SARS-CoV-2. Contact lenses with nanoparticles releasing griffithsin may be a way to protect the ocular surface from SARS-CoV-2 infection and provide a supplementary protection method for health care workers in daily practice.

## Discussion

The global pandemic of SARS-CoV-2 in 2020 has caused tremendous pressure on the health systems of almost every country in the world. Due to inappropriate protection and shortage of medical supplies, many medical staffs got infected (47). SARS-CoV-2 has relatively high infectivity and mainly spreads *via* close contact and droplets. There is ongoing hot debate on the potential role of ocular surface in the transmission of SARS-CoV-2, and some clinical and laboratory findings support that ocular involvement was observed in a minority of patients. For medical workers with high risk of aerosol exposure and close contact with patients, ocular surface may be a potential and overlooked site of contamination. WHO has alarmed medical stuffs to wear protective goggles during the whole contact period with patients who were suspected or confirmed to be infected (48).

However, for daily medical practice in ordinary clinics, wearing eye goggles is not mandatory or always practical. Although eye goggles seem to provide the best protection and not harmful to ocular surface, they have several disadvantages. First of all, goggles are generally uncomfortable to use, and very likely to disturb vision due to extensive formation of water droplets. Thus protective goggles are very inconvenient for doctors who require precise vision, including ophthalmologists, dentists, surgeons and so on. Besides, long-term use of eye goggles is reported to disturb working status and may lead to increased medical errors. In a recent survey conducted during SARS-CoV-2 outbreak on 231 nurses in China, use of eye goggles caused headache, skin pressure injury and dizziness in 79%, 66%, and 49% of nurses, respectively. 82.7% of nurses subjectively reported that use of eye goggles negatively impacted their working status, and events of medical errors were reported in 19.5% of nurses wearing goggles (49). Third, foggy goggles may interfere with vision and need frequent adjustment during use, which was reported in 59.7% of nurses in China (49). The adjustment may lead to increased risk of being infected. In addition, SARS-CoV-2 infection due to dislocation of eye goggles has also been reported in an emergency nurse (17). Due to long incubation period and relatively high proportion of asymptomatic infection of SARS-CoV-2, it is difficult to identify infected patients in the beginning (50). So during the outbreak period, any medical workers are at risk of being infected, because they may be likely to contact closely with an asymptomatic patient in the outpatient clinics or during physical examinations. For example, during the slit lamp or direct fundoscopy examination, an ophthalmology doctor need to directly face the patient at a distance of 3–10 cm. There is also huge risk of aerosol exposure during processes such as dental repair, open surgery, tracheal intubation and so on (51, 52). A recent survey conducted in British ophthalmology practitioners showed that they were very unconfident about no ocular protection in the daily work and called for more eye protection (53). Thus, it is necessary to provide adequate eye protection for medical workers during the outbreak period, as medical workers are at higher risk of aerosol exposure which can potentially result in risk of ocular contamination.

Based on the broad spectrum antiviral activity of griffithsin, we proposed a theoretical device of contact lenses with griffithsin nanoparticles as a potential alternative personal protective equipment against SARS-CoV-2. Although no previous data of the antiviral efficacy of griffithsin on SARS-CoV-2 is available, we made the hypothesis based on the efficacy study of griffithsin on other common viruses, including MERS-CoV, SARS-CoV, HIV, HCV, and so on (29–32). Griffithsin is continuously released onto the ocular surface and can bind directly to the S protein of coronavirus to block the entry of virus. The sustained releasing system enables prolonged protection time. Besides, contact lens doesn't disturb vision and is relatively well-tolerated by regular users. It can be served as voluntary choice for those who tolerate well and need precise vision during clinical practice. Based on current available results, ocular involvement is found in a small number of patients confirmed to be infected by SARS-CoV-2. We consider the ocular surface is likely to be a minor transmission route, so contact lenses containing griffithsin may provide sufficient protection for medical workers not directly facing high risk patients. Besides, as griffithsin has anti-viral activity against a broad spectrum of enveloped virus, this therapeutic contact lenses can be further applied in a variety of situations which require eye protection for medical practitioners. In addition, Decker et al. proposed a low cost lab-scale production method of griffithsin with engineered *E. coli*, which could generate more than 20 tons of griffithsin per year at the cost of below 3,500$ (42). This would make the griffithsin-loaded contact lens affordable to the medical systems.

Despite the potential benefit for griffithsin-loaded contact lens to act against ocular transmission of SARS-CoV-2, special attention should be paid to the safety concerns associated with contact lens wear. Incidence of infectious keratitis, Acanthamoeba and fungal infections related to contact lens use is on the rise in recent years (54). According to a survey of contact lens users in USA, nearly a third of them reported previous contact lens-related red or painful eye requiring a doctor's visit (55). Thus, infection risk is a potential limitation for our proposed protection method. However, several ways can be taken to control the risk of bacterial keratitis. First of all, griffithsin-loaded contact lens is basically designed for health care workers, who generally have higher awareness of the importance of hand hygiene before applying (56). Second, the contact lens should be designed as daily disposable use to reduce infection associated with overnight wear, long-term use and case pollution (57, 58). As reported in a study in Australia, the rate of microbial keratitis associated with daily disposable contact lens wear is relatively low (1–2 per 10,000 wears per year) (59).

As therapeutic contact lens can only cover the corneal portion of the eye, there is potential risk of uncovered part to be infected. However, griffithsin can dissolve into tear film and spread over the ocular surface. This will expand its protection area beyond the covered part. Besides, as shown in previous studies, griffithsin is a highly potent antiviral agent and is effective at very low concentration, which indicates that griffithsin dissolved in tear film may also have antiviral activity (34, 36). As for SARS-CoV-2, no data of inhibition efficacy is currently available. Thus, pharmacokinetic studies of tear concentration after application of the therapeutic contact lens need to be compared with the antiviral concentration of SARS-CoV-2 in order to decide the longest protection time.

Another potential limitation of griffithsin-loaded contact lens is its potential ocular toxicity associated with the medication. Although lectin is commonly used in ocular formulation to improve drug retention time, currently no ocular formulation and safety profile of griffithsin on ocular tissues is available. As indicated in the inhibition study of SARS-CoV, griffithsin is multivalent and can effectively inhibit the virus at low concentration of 0.28–0.96 μM. A previous safety study showed that mucosal or systemic administration of 2 mg/kg griffithsin on mice should no systemic toxicity *in vivo* (60). An *in vitro* study showed that compared with other anti-viral lectins, application of griffithsin showed minimal effects of toxicity, T cell activation and alteration of gene expressions, which indicated excellent safety profile (41). To date, the safety of griffithsin has been tested in two phase 1 clinical trials on human (NCT04032717 and NCT02875119), but the results have not been published. In the two clinical trials, griffithsin was applied as either vaginal gel (at variable doses) or rectal enema (4.2 ml in volume containing 9.6 mg/ml of griffithsin) to prevent HIV-1 infection. As ocular surface is a special area and more sensitive to drug irritation, more *in vitro* and *in vivo* preclinical studies on the ocular safety of different doses of griffithsin are preliminarily required. The safety issues regarding long-term ocular application of griffithsin *via* contact lens need to be verified and the concentration of griffithsin need to be set at minimal inhibition concentration in order to avoid supratherapeutic toxicity. The protection benefits and potential adverse effects of griffithsin-loaded contact lens should be balanced and considered before applying for use in clinics.

Overall, griffithsin-loaded contact lens can be considered as a supplementary choice for ocular protection besides eye goggles for health care workers during SARS-CoV-2 outbreak.

## Data Availability Statement

The original contributions presented in the study are included in the article/supplementary material, further inquiries can be directed to the corresponding author/s.

## Author Contributions

LW conducted literature search. YD provided guidance and approved the final manuscript. Both authors proposed and discussed about the idea of the hypothesis and contributed to manuscript writing and editing.

## Conflict of Interest

The authors declare that the research was conducted in the absence of any commercial or financial relationships that could be construed as a potential conflict of interest.

## References

[1] Wan Y Shang J Graham R Baric RS Li F. Receptor recognition by novel coronavirus from Wuhan: an analysis based on decade-long structural studies of SARS. J Virol. (2020) 7:e00127-20. 10.1128/JVI.00127-20 31996437 PMC7081895

[2] Wang K Chen W Zhou YS Lian JQ Zhang Z Du P SARS-CoV-2 invades host cells via a novel route: CD147-spike protein. bioRxiv [Preprint]. (2020). 10.1101/2020.03.14.988345

[3] Shang J Wan Y Luo C Ye G Geng Q Auerbach A Cell entry mechanisms of SARS-CoV-2. Proc Natl Acad Sci USA. (2020) 117:11727–34. 10.1073/pnas.2003138117 32376634 PMC7260975

[4] Hoffmann M Kleine-Weber H Schroeder S Krüger N Herrler T Erichsen S SARS-CoV-2 cell entry depends on ACE2 and TMPRSS2 and is blocked by a clinically proven protease inhibitor. Cell. (2020) 181:271–80.e8. 10.1016/j.cell.2020.02.052 32142651 PMC7102627

[5] Zhou L Xu Z Castiglione GM Soiberman US Eberhart CG Duh EJ. ACE2 and TMPRSS2 are expressed on the human ocular surface, suggesting susceptibility to SARS-CoV-2 infection. Ocul Surf. (2020) 18:537–44. 10.1016/j.jtos.2020.06.007 32544566 PMC7293510

[6] Määttä M Tervahartiala T Kaarniranta K Tang Y Yan L Tuukkanen J. Immunolocalization of EMMPRIN (CD147) in the human eye and detection of soluble form of EMMPRIN in ocular fluids. Curr Eye Res. (2006) 31:917–24. 10.1080/02713680600932290 17114117

[7] Collin J Queen R Zerti D Dorgau B Georgiou M Djidrovski I. Co-expression of SARS-CoV-2 entry genes in the superficial adult human conjunctival, limbal and corneal epithelium suggests an additional route of entry via the ocular surface. Ocul Surf. (2020). [Epub ahead of print]. 10.1016/j.jtos.2020.05.013 32502616 PMC7267807

[8] Duan M Liu L Da G Gehin E Nielsen PV Weinreich UM. Measuring the administered dose of particles on the facial mucosa of a realistic human model. Indoor Air. (2020) 30:108–16. 10.1111/ina.12612 31608493

[9] Napoli PE Nioi M d'Aloja E Fossarello M. The ocular surface and the coronavirus disease 2019: does a dual 'Ocular Route' exist? J Clin Med. (2020) 9:1269. 10.3390/jcm9051269 32353982 PMC7287662

[10] Seah I Agrawal R. Can the Coronavirus disease 2019 (COVID-19) affect the eyes? a review of coronaviruses and ocular implications in humans and animals. Ocul Immunol Inflamm. (2020) 28:391–5. 10.1080/09273948.2020.1738501 32175797 PMC7103678

[11] Coen M Allali G Adler D Serratrice J. Hypoxemia in COVID-19; comment on: “The neuroinvasive potential of SARS - CoV2 may play a role in the respiratory failure of COVID-19 patients”. J Med Virol. (2020). [Epub ahead of print]. 10.1002/jmv.26020 32418237 PMC7276823

[12] Deng W Bao L Gao H Xiang Z Qu Y Song Z. Ocular conjunctival inoculation of SARS-CoV-2 can cause mild COVID-19 in rhesus macaques. Nat Commun. (2020) 11:4400. 10.1038/s41467-020-18149-6 32879306 PMC7467924

[13] Zhou Y Zeng Y Tong Y Chen C Ophthalmologic evidence against the interpersonal transmission of 2019 novel coronavirus through conjunctiva. medRxiv. [Preprint]. (2020). 10.1101/2020.02.11.20021956

[14] Chen L Deng C Chen X Zhang X Chen B Yu H. Ocular manifestations and clinical characteristics of 535 cases of COVID-19 in Wuhan, China: a cross-sectional study. Acta Ophthalmol. (2020). [Epub ahead of print]. 10.1111/aos.14472 32421258 PMC7276826

[15] Wu P Duan F Luo C Liu Q Qu X Liang L. Characteristics of ocular findings of patients with coronavirus disease 2019 (COVID-19) in Hubei Province, China. JAMA Ophthalmol. (2020) 138:575–8. 10.1001/jamaophthalmol.2020.1291 32232433 PMC7110919

[16] Xia J Tong J Liu M Shen Y Guo D. Evaluation of coronavirus in tears and conjunctival secretions of patients with SARS-CoV-2 infection. J Med Virol. (2020) 92:589–94. 10.1002/jmv.25725 32100876 PMC7228294

[17] Zhang X Chen X Chen L Deng C Zou X Liu W. The evidence of SARS-CoV-2 infection on ocular surface. Ocul Surf. (2020) 18:360–2. 10.1016/j.jtos.2020.03.010 32289466 PMC7194535

[18] Xu L Zhang X Song W Sun B Mu J Dong X Conjunctival polymerase chain reaction-tests of 2019 novel coronavirus in patients in Shenyang,China. medRxiv. [Preprint]. (2020). 10.1101/2020.02.23.20024935

[19] Fang Z Zhang Y Hang C Zhang W Ai J Li S. Comparisons of nucleic acid conversion time of SARS-CoV-2 of different samples in ICU and non-ICU patients. J Infect. (2020) 81:147–78. 10.1016/j.jinf.2020.03.013 32209381 PMC7118636

[20] Deng C Yang Y Chen H Chen W Chen Z ma K Ocular dectection of SARS-CoV-2 in 114 cases of COVID-19 pneumonia in Wuhan, China: an observational study. Lancet. [Preprint]. (2020) 10.2139/ssrn.3543587

[21] Aiello F Gallo Afflitto G Mancino R Li JO Cesareo M Giannini C. Coronavirus disease 2019 (SARS-CoV-2) and colonization of ocular tissues and secretions: a systematic review. Eye. (2020) 34:1206–11. 10.1038/s41433-020-0926-9 32424327 PMC7232062

[22] Zimmerman K Kearns F Tzekov R. Natural protection of ocular surface from viral infections - A hypothesis. Med Hypotheses. (2020) 143:110082. 10.1016/j.mehy.2020.110082 32679424 PMC7346787

[23] Akpek EK Gottsch JD. Immune defense at the ocular surface. Eye. (2003) 17:949–56. 10.1038/sj.eye.6700617 14631402

[24] Mick P Murphy R. Aerosol-generating otolaryngology procedures and the need for enhanced PPE during the COVID-19 pandemic: a literature review. J Otolaryngol. (2020) 49:29. 10.1186/s40463-020-00424-7 32393346 PMC7212733

[25] Napoli PE Nioi M d'Aloja E Fossarello M. Safety recommendations and medical liability in ocular surgery during the COVID-19 pandemic: an unsolved dilemma. J Clin Med. (2020) 9:1403. 10.3390/jcm9051403 32397530 PMC7290727

[26] Raboud J Shigayeva A McGeer A Bontovics E Chapman M Gravel D. Risk factors for SARS transmission from patients requiring intubation: a multicentre investigation in Toronto, Canada. PLoS ONE. (2010) 5:e10717. 10.1371/journal.pone.0010717 20502660 PMC2873403

[27] American Academy of Ophthalmology. Important coronavirus updates for ophthalmologists (2020). Available online at: https://www.aao.org/headline/alert-important-coronavirus-context (accessed March 23, 2020).

[28] Li L Tian X Chen J Li P Zheng Q Hou J. Griffithsin inhibits porcine reproductive and respiratory syndrome virus infection *in vitro*. Arch Virol. (2018) 163:3317–25. 10.1007/s00705-018-4029-x 30220033 PMC7087274

[29] Xue J Gao Y Hoorelbeke B Kagiampakis I Zhao B Demeler B. The role of individual carbohydrate-binding sites in the function of the potent anti-HIV lectin griffithsin. Mol Pharm. (2012) 9:2613–25. 10.1021/mp300194b 22827601 PMC3474606

[30] Takebe Y Saucedo CJ Lund G Uenishi R Hase S Tsuchiura T. Antiviral lectins from red and blue-green algae show potent *in vitro* and *in vivo* activity against hepatitis C virus. PLoS ONE. (2013) 8:e64449. 10.1371/journal.pone.0064449 23700478 PMC3660260

[31] Millet JK Séron K Labitt RN Danneels A Palmer KE Whittaker GR. Middle East respiratory syndrome coronavirus infection is inhibited by griffithsin. Antiviral Res. (2016) 133:1–8. 10.1016/j.antiviral.2016.07.011 27424494 PMC7113895

[32] O'Keefe BR Giomarelli B Barnard DL Shenoy SR Chan PK McMahon JB. Broad-spectrum *in vitro* activity and *in vivo* efficacy of the antiviral protein griffithsin against emerging viruses of the family Coronaviridae. J Virol. (2010) 84:2511–21. 10.1128/JVI.02322-09 20032190 PMC2820936

[33] Lusvarghi S Bewley CA. Griffithsin: an antiviral lectin with outstanding therapeutic potential. Viruses. (2016) 8:296. 10.3390/v8100296 27783038 PMC5086628

[34] Emau P Tian B O'Keefe B R Mori T McMahon JB Palmer KE. Griffithsin, a potent HIV entry inhibitor, is an excellent candidate for anti-HIV microbicide. J Med Primatol. (2007) 36:244–53. 10.1111/j.1600-0684.2007.00242.x 17669213

[35] Nixon B Stefanidou M Mesquita PM Fakioglu E Segarra T Rohan L. Griffithsin protects mice from genital herpes by preventing cell-to-cell spread. J Virol. (2013) 87:6257–69. 10.1128/JVI.00012-13 23536670 PMC3648100

[36] Ziółkowska NE O'Keefe BR Mori T Zhu C Giomarelli B Vojdani F. Domain-swapped structure of the potent antiviral protein griffithsin and its mode of carbohydrate binding. Structure. (2006) 14:1127–35. 10.1016/j.str.2006.05.017 16843894 PMC7126681

[37] Ahmed SF Quadeer AA McKay MR. Preliminary identification of potential vaccine targets for the COVID-19 coronavirus (SARS-CoV-2) based on SARS-CoV immunological studies. Viruses. (2020) 12:254. 10.3390/v12030254 32106567 PMC7150947

[38] Zhou D Tian X Qi R Peng C Zhang W. Identification of 22 N-glycosites on spike glycoprotein of SARS-CoV-2 and accessible surface glycopeptide motifs: implications for vaccination and antibody therapeutics. Glycobiology. (2020) 30:cwaa052. 10.1093/glycob/cwaa052 32518941 PMC7313968

[39] Han DP Kim HG Kim YB Poon LL Cho MW. Development of a safe neutralization assay for SARS-CoV and characterization of S-glycoprotein. Virology. (2004) 326:140–9. 10.1016/j.virol.2004.05.017 15262502 PMC7127165

[40] Moncla BJ Pryke K Rohan LC Graebing PW. Degradation of naturally occurring and engineered antimicrobial peptides by proteases. Adv Biosci Biotechnol. (2011) 2:404–8. 10.4236/abb.2011.26059 22611520 PMC3354962

[41] Kouokam JC Huskens D Schols D Johannemann A Riedell SK Walter W. Investigation of griffithsin's interactions with human cells confirms its outstanding safety and efficacy profile as a microbicide candidate. PLoS ONE. (2011) 6:e22635. 10.1371/journal.pone.0022635 21829638 PMC3149051

[42] Decker JS Menacho-Melgar R Lynch MD Low-cost large-scale production of the anti-viral lectin griffithsin Front Bioeng Biotechnol. (2020) 8:1020 10.3389/fbioe.2020.01020 32974328 PMC7471252

[43] Lotfi M Hamblin MR Rezaei N. COVID-19: transmission, prevention, and potential therapeutic opportunities. Clin Chim Acta. (2020) 508:254–66. 10.1016/j.cca.2020.05.044 32474009 PMC7256510

[44] Urtti A. Challenges and obstacles of ocular pharmacokinetics and drug delivery. Adv Drug Delivery Rev. (2006) 58:1131–5. 10.1016/j.addr.2006.07.027 17097758

[45] Gulsen D Chauhan A. Ophthalmic drug delivery through contact lenses. Invest Ophthalmol Visual Sci. (2004) 45:2342–7. 10.1167/iovs.03-0959 15223815

[46] Maulvi FA Soni TG Shah DO. A review on therapeutic contact lenses for ocular drug delivery. Drug Delivery. (2016) 23:3017–26. 10.3109/10717544.2016.1138342 26821766

[47] Ranney ML Griffeth V Jha AK. Critical supply shortages - the need for ventilators and personal protective equipment during the Covid-19 pandemic. N Engl J Med. (2020) 382:e41. 10.1056/NEJMp2006141 32212516

[48] World Health Organization Rational use of personal protective equipment (PPE) for coronavirus disease (COVID-19): interim guidance. (2020). Available online at: https://apps.who.int/iris/handle/10665/331215 (accessed February 27, 2020).

[49] He XH Feng YR Li GM Pang XJ Chen T Zhou YL The impact of goggle-associated harms to health and working status of nurses during management of COVID-19. medRxiv. [Preprint]. (2020). 10.1101/2020.05.11.20094854

[50] Lai CC Liu YH Wang CY Wang YH Hsueh SC Yen MY. Asymptomatic carrier state, acute respiratory disease, and pneumonia due to severe acute respiratory syndrome coronavirus 2 (SARS-CoV-2): facts and myths. J Microbiol Immunol Infect. (2020) 53:404–12. 10.1016/j.jmii.2020.02.012 32173241 PMC7128959

[51] Zemouri C. de Soet H Crielaard W Laheij A. A scoping review on bio-aerosols in healthcare and the dental environment. PLoS ONE. (2017) 12:e0178007. 10.1371/journal.pone.0178007 28531183 PMC5439730

[52] Bing Y Zhang YH Leung NHL Cowling BJ Yang ZF. Role of viral bioaerosols in nosocomial infections and measures for prevention and control. J Aerosol Sci. (2018) 117:200–11. 10.1016/j.jaerosci.2017.11.011 32226118 PMC7094610

[53] Minocha A Sim SY Than J Vakros G. Survey of ophthalmology practitioners in A&E on current COVID-19 guidance at three major UK eye hospitals. Eye. (2020) 34:1243–5. 10.1038/s41433-020-0857-5 32265510 PMC7136748

[54] Cheung N Nagra P Hammersmith K. Emerging trends in contact lens-related infections. Curr Opin Ophthalmol. (2016) 27:327–32. 10.1097/ICU.0000000000000280 27176217

[55] Cope JR Collier SA Rao MM Chalmers R Mitchell GL Richdale K. Contact lens wearer demographics and risk behaviors for contact lens-related eye infections–United States, 2014. MMWR Morb Mortal Wkly Rep. (2015) 64:865–70. 10.15585/mmwr.mm6432a2 26292204 PMC5779588

[56] Stapleton F Naduvilath T Keay L Radford C Dart J Edwards K. Risk factors and causative organisms in microbial keratitis in daily disposable contact lens wear. PLoS ONE. (2017) 12:e0181343. 10.1371/journal.pone.0181343 28813424 PMC5558933

[57] Lim CH Carnt NA Farook M Lam J Tan DT Mehta JS. Risk factors for contact lens-related microbial keratitis in Singapore. Eye. (2016) 30:447–55. 10.1038/eye.2015.250 26634710 PMC4791703

[58] Carnt N Stapleton F. Strategies for the prevention of contact lens-related *Acanthamoeba keratitis*: a review. Ophthalmic Physiol Opt. (2016) 36:77–92. 10.1111/opo.12271 26691018

[59] Stapleton F Keay L Edwards K Naduvilath T Dart JK Brian G. The incidence of contact lens-related microbial keratitis in Australia. Ophthalmology. (2008) 115:1655–62. 10.1016/j.ophtha.2008.04.002 18538404

[60] Kouokam JC Lasnik AB Palmer KE. Studies in a murine model confirm the safety of griffithsin and advocate its further development as a microbicide targeting HIV-1 and other enveloped viruses. Viruses. (2016) 8:311. 10.3390/v8110311 27869695 PMC5127025

